# Tetra-μ-benzoato-bis­[(quinoxaline)copper(II)]

**DOI:** 10.1107/S1600536807067876

**Published:** 2008-01-04

**Authors:** Eun Yong Lee, Byeong Kwon Park, Cheal Kim, Sung-Jin Kim, Youngmee Kim

**Affiliations:** a Department of Fine Chemistry, Eco-Product and Materials Education Center, Seoul National University of Technology, Seoul 139-743, Republic of Korea; b Division of Nano Sciences, Ewha Womans University, Seoul 120-750, Republic of Korea

## Abstract

The paddlewheel-type centrosymmetric dinuclear title complex, [Cu_2_(C_7_H_5_O_2_)_4_(C_8_H_6_N_2_)_2_], contains four bridging benzoate groups and two terminal quinoxaline ligands. The octa­hedral coordination around each Cu atom, with four O atoms in the equatorial plane, is completed by an N atom of a quinoxaline mol­ecule [Cu—N = 2.2465 (18) Å] and by the second Cu atom [Cu⋯Cu = 2.668 (5) Å]. The Cu atom is 0.216 Å out of the plane of the four O atoms.

## Related literature

For the related structure, Cu_2_(O_2_CPh)_4_(py)_2_ (py = pyridine), see: Speier & Fülöp (1989 ▶). For background information, see: Cotton & Walton (1993 ▶); Pichon *et al.* (2007 ▶); Goto *et al.* (2007 ▶); Takamizawa *et al.* (2004 ▶); Casarin *et al.* (2005 ▶); Deka *et al.* (2006 ▶).
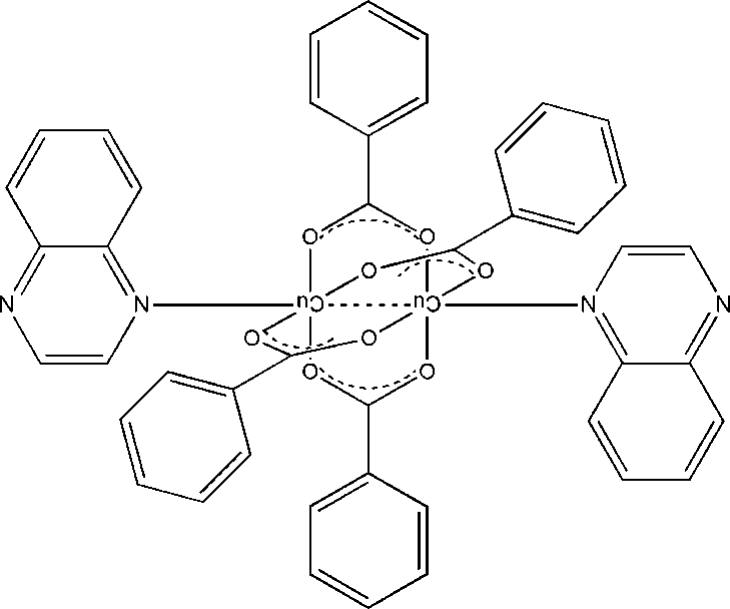


## Experimental

### 

#### Crystal data

[Cu_2_(C_7_H_5_O_2_)_4_(C_8_H_6_N_2_)_2_]
*M* *_r_* = 871.82
Triclinic, 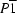

*a* = 10.1423 (16) Å
*b* = 10.3400 (17) Å
*c* = 10.5148 (17) Å
α = 65.459 (2)°
β = 73.063 (3)°
γ = 82.142 (3)°
*V* = 959.4 (3) Å^3^
*Z* = 1
Mo *K*α radiation
μ = 1.17 mm^−1^
*T* = 293 (2) K
0.15 × 0.10 × 0.08 mm

#### Data collection

Bruker SMART CCD area-detector diffractometer
Absorption correction: none
5377 measured reflections
3668 independent reflections
2983 reflections with *I* > 2σ(*I*)
*R*_int_ = 0.031

#### Refinement

*R*[*F* ^2^ > 2σ(*F* ^2^)] = 0.033
*wR*(*F* ^2^) = 0.080
*S* = 0.96
3668 reflections
262 parameters
H-atom parameters constrained
Δρ_max_ = 0.28 e Å^−3^
Δρ_min_ = −0.41 e Å^−3^

### 

Data collection: *SMART* (Bruker, 1997 ▶); cell refinement: *SAINT* (Bruker, 1997 ▶); data reduction: *SAINT*; program(s) used to solve structure: *SHELXS97* (Sheldrick, 1997 ▶); program(s) used to refine structure: *SHELXL97* (Sheldrick, 1997 ▶); molecular graphics: *SHELXTL* (Bruker, 1998 ▶); software used to prepare material for publication: *SHELXTL*.

## Supplementary Material

Crystal structure: contains datablocks I, global. DOI: 10.1107/S1600536807067876/dn2301sup1.cif

Structure factors: contains datablocks I. DOI: 10.1107/S1600536807067876/dn2301Isup2.hkl

Additional supplementary materials:  crystallographic information; 3D view; checkCIF report

## Figures and Tables

**Fig. 1.**
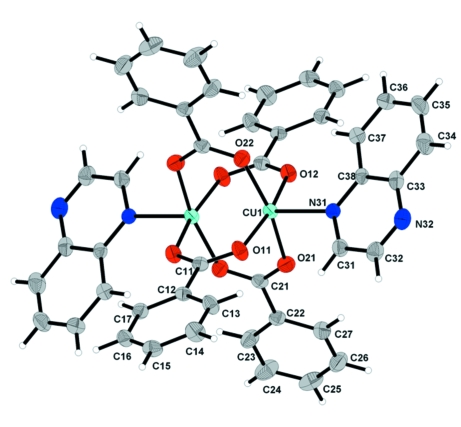
The structure of the title compound showing the atom-labeling scheme. Displacement ellipsoids are shown at the 30% probability level.

## supplementary crystallographic information

### Comment

The dinuclear metal carboxylates, *M*_2_(O_2_CR)_4_, are important for the study of structures and metal-metal interaction (Cotton & Walton, 1993). Among them copper(II) carboxylates are used as building blocks to form a pillard-grid MOF with large pores (Pichon *et al.*, 2007), and copper(II) benzoate pyrazine is used as the organic-inorganic hybrid complex that adsorbs gas molecules through clathrate formation (Goto *et al.*, 2007, Takamizawa *et al.*, 2004). Due to different coordination modes of carboxylates (Casarin *et al.*, 2005), it is essential to have control on the binding of carboxylate to a metal ion in specific manner in the presence of other ligands (Deka *et al.*, 2006). Controlling the binding of carboxylate will make it possible to synthesize complexes having new structures. We report here on the structure of new copper(II) benzoate with quinoxaline.

Asymmetric unit contains half of whole molecule, and there is an inversion center in the middle of Cu—Cu bond. Symmetric operation (-*x* + 2, -*y* + 1, -*z* + 1) produces a paddle-wheel type dinuclear copper-benzoate complex (Fig. 1). The paddle-wheel type dinuclear complex is constructed by four bridging benzoate groups and two terminal quinoxaline ligands. The octahedral coordination around the copper atom is completed by nitrogen atom of quinoxaline molecule (Cu—N 2.2465 (18) Å) and by the second copper atom (Cu···Cu 2.668 (5) Å). The copper atom is 0.216 Å out of the plane of the four oxygen atoms.

### Experimental

19.0 mg (0.1 mmol) of Cu(NO_3_)_2_.2.5H_2_O and 28.0 mg (0.2 mmol) of C_6_H_5_COONH_4_ were dissolved in 4 ml me thanol and carefully layered by 4 ml acetone solution of quinoxaline ligand (26.0 mg, 0.2 mmol). Suitable crystals of the title compound for X-ray analysis were obtained in a few weeks.

### Refinement

H atoms were placed in calculated positions with C—H distances of 0.93 Å. They were included in the refinement in riding-motion approximation with *U*_iso_(H) = 1.2*U*_eq_(C).

### Crystal data

**Table:**

[Cu_2_(C_7_H_5_O_2_)_4_(C_8_H_6_N_2_)_2_]	*Z* = 1
*M**_r_* = 871.82	*F*_000_ = 446
Triclinic, *P*1	*D*_x_ = 1.509 Mg m^−^^3^
Hall symbol: -P 1	Mo *K*α radiation λ = 0.71073 Å
*a* = 10.1423 (16) Å	Cell parameters from 2414 reflections
*b* = 10.3400 (17) Å	θ = 2.4–27.2º
*c* = 10.5148 (17) Å	µ = 1.17 mm^−^^1^
α = 65.459 (2)º	*T* = 293 (2) K
β = 73.063 (3)º	Block, blue
γ = 82.142 (3)º	0.15 × 0.10 × 0.08 mm
*V* = 959.4 (3) Å^3^	

### Data collection

**Table:**

Bruker SMART CCD area-detector diffractometer	2983 reflections with *I* > 2σ(*I*)
Radiation source: fine-focus sealed tube	*R*_int_ = 0.031
Monochromator: graphite	θ_max_ = 26.0º
*T* = 293(2) K	θ_min_ = 2.1º
φ and ω scans	*h* = −6→12
Absorption correction: none	*k* = −12→12
5377 measured reflections	*l* = −11→12
3668 independent reflections	

### Refinement

**Table:**

Refinement on *F*^2^	Secondary atom site location: difference Fourier map
Least-squares matrix: full	Hydrogen site location: inferred from neighbouring sites
*R*[*F*^2^ > 2σ(*F*^2^)] = 0.033	H-atom parameters constrained
*wR*(*F*^2^) = 0.080	*w* = 1/[σ^2^(*F*_o_^2^) + (0.0403*P*)^2^] where *P* = (*F*_o_^2^ + 2*F*_c_^2^)/3
*S* = 0.96	(Δ/σ)_max_ = 0.002
3668 reflections	Δρ_max_ = 0.28 e Å^−^^3^
262 parameters	Δρ_min_ = −0.41 e Å^−^^3^
Primary atom site location: structure-invariant direct methods	Extinction correction: none

### Special details

**Table:**

Geometry. All e.s.d.'s (except the e.s.d. in the dihedral angle between two l.s. planes) are estimated using the full covariance matrix. The cell e.s.d.'s are taken into account individually in the estimation of e.s.d.'s in distances, angles and torsion angles; correlations between e.s.d.'s in cell parameters are only used when they are defined by crystal symmetry. An approximate (isotropic) treatment of cell e.s.d.'s is used for estimating e.s.d.'s involving l.s. planes.
Refinement. Refinement of *F*^2^ against ALL reflections. The weighted *R*-factor *wR* and goodness of fit *S* are based on *F*^2^, conventional *R*-factors *R* are based on *F*, with *F* set to zero for negative *F*^2^. The threshold expression of *F*^2^ > σ(*F*^2^) is used only for calculating *R*-factors(gt) *etc*. and is not relevant to the choice of reflections for refinement. *R*-factors based on *F*^2^ are statistically about twice as large as those based on *F*, and *R*- factors based on ALL data will be even larger.

### Fractional atomic coordinates and isotropic or equivalent isotropic displacement parameters (Å^2^)

**Table:**

	*x*	*y*	*z*	*U*_iso_*/*U*_eq_	
Cu1	1.11111 (3)	0.47628 (3)	0.40624 (3)	0.03419 (11)	
O11	0.99508 (16)	0.32526 (17)	0.43180 (19)	0.0499 (5)	
O12	1.18983 (16)	0.63828 (16)	0.40754 (17)	0.0440 (4)	
C11	0.8752 (2)	0.2967 (2)	0.5137 (2)	0.0369 (5)	
C12	0.8048 (2)	0.1757 (2)	0.5189 (2)	0.0351 (5)	
C13	0.8728 (3)	0.0988 (3)	0.4383 (3)	0.0492 (6)	
H13	0.9618	0.1230	0.3800	0.059*	
C14	0.8095 (3)	−0.0143 (3)	0.4433 (3)	0.0568 (7)	
H14	0.8564	−0.0666	0.3897	0.068*	
C15	0.6776 (3)	−0.0490 (3)	0.5274 (3)	0.0498 (7)	
H15	0.6349	−0.1247	0.5306	0.060*	
C16	0.6087 (3)	0.0279 (3)	0.6069 (3)	0.0470 (6)	
H16	0.5191	0.0044	0.6637	0.056*	
C17	0.6716 (2)	0.1398 (2)	0.6030 (2)	0.0408 (6)	
H17	0.6243	0.1916	0.6572	0.049*	
O21	1.02164 (17)	0.60977 (18)	0.25640 (18)	0.0495 (4)	
O22	1.16461 (17)	0.35143 (16)	0.58557 (17)	0.0452 (4)	
C21	0.9089 (2)	0.6714 (2)	0.2873 (2)	0.0370 (5)	
C22	0.8591 (2)	0.7841 (2)	0.1645 (2)	0.0382 (5)	
C23	0.7344 (3)	0.8541 (3)	0.1901 (3)	0.0535 (7)	
H23	0.6771	0.8249	0.2837	0.064*	
C24	0.6936 (3)	0.9660 (3)	0.0797 (3)	0.0694 (9)	
H24	0.6096	1.0123	0.0993	0.083*	
C25	0.7761 (4)	1.0094 (3)	−0.0586 (3)	0.0678 (9)	
H25	0.7497	1.0866	−0.1329	0.081*	
C26	0.8975 (3)	0.9385 (3)	−0.0869 (3)	0.0666 (8)	
H26	0.9523	0.9660	−0.1815	0.080*	
C27	0.9401 (3)	0.8263 (3)	0.0233 (3)	0.0540 (7)	
H27	1.0232	0.7791	0.0026	0.065*	
N31	1.28562 (19)	0.41356 (19)	0.25284 (19)	0.0354 (4)	
N32	1.4763 (2)	0.2919 (2)	0.0746 (2)	0.0522 (6)	
C31	1.2485 (3)	0.3591 (3)	0.1760 (3)	0.0457 (6)	
H31	1.1554	0.3601	0.1804	0.055*	
C32	1.3443 (3)	0.2991 (3)	0.0868 (3)	0.0519 (7)	
H32	1.3115	0.2631	0.0344	0.062*	
C33	1.5184 (2)	0.3494 (2)	0.1517 (3)	0.0443 (6)	
C34	1.6606 (3)	0.3475 (3)	0.1428 (3)	0.0615 (8)	
H34	1.7237	0.3059	0.0862	0.074*	
C35	1.7047 (3)	0.4059 (3)	0.2163 (3)	0.0684 (9)	
H35	1.7984	0.4048	0.2089	0.082*	
C36	1.6121 (3)	0.4678 (3)	0.3030 (3)	0.0608 (8)	
H36	1.6448	0.5078	0.3523	0.073*	
C37	1.4736 (3)	0.4702 (3)	0.3163 (3)	0.0468 (6)	
H37	1.4124	0.5106	0.3753	0.056*	
C38	1.4240 (2)	0.4112 (2)	0.2406 (2)	0.0363 (5)	

### Atomic displacement parameters (Å^2^)

**Table:**

	*U*^11^	*U*^22^	*U*^33^	*U*^12^	*U*^13^	*U*^23^
Cu1	0.03083 (17)	0.03639 (17)	0.04054 (18)	−0.00078 (11)	−0.00605 (12)	−0.02234 (13)
O11	0.0379 (10)	0.0541 (11)	0.0676 (12)	−0.0115 (8)	0.0016 (9)	−0.0407 (9)
O12	0.0422 (10)	0.0443 (10)	0.0528 (10)	−0.0082 (8)	−0.0025 (8)	−0.0307 (8)
C11	0.0375 (14)	0.0357 (13)	0.0414 (13)	−0.0001 (11)	−0.0138 (12)	−0.0168 (11)
C12	0.0380 (13)	0.0327 (12)	0.0396 (13)	−0.0006 (10)	−0.0150 (11)	−0.0159 (10)
C13	0.0387 (14)	0.0505 (15)	0.0651 (17)	−0.0080 (12)	−0.0026 (13)	−0.0344 (14)
C14	0.0577 (18)	0.0534 (17)	0.0745 (19)	−0.0017 (14)	−0.0120 (15)	−0.0430 (15)
C15	0.0589 (18)	0.0380 (14)	0.0593 (16)	−0.0103 (12)	−0.0237 (14)	−0.0174 (12)
C16	0.0423 (15)	0.0473 (15)	0.0485 (15)	−0.0119 (12)	−0.0099 (12)	−0.0141 (12)
C17	0.0433 (14)	0.0411 (14)	0.0403 (13)	−0.0041 (11)	−0.0112 (11)	−0.0172 (11)
O21	0.0420 (10)	0.0636 (11)	0.0481 (10)	0.0139 (9)	−0.0157 (8)	−0.0291 (9)
O22	0.0435 (10)	0.0467 (10)	0.0421 (10)	0.0063 (8)	−0.0103 (8)	−0.0171 (8)
C21	0.0380 (14)	0.0378 (13)	0.0463 (14)	−0.0037 (11)	−0.0137 (12)	−0.0246 (11)
C22	0.0433 (14)	0.0392 (13)	0.0408 (13)	−0.0027 (11)	−0.0134 (11)	−0.0220 (11)
C23	0.0600 (18)	0.0604 (17)	0.0413 (14)	0.0139 (14)	−0.0151 (13)	−0.0246 (13)
C24	0.089 (2)	0.067 (2)	0.0587 (19)	0.0328 (18)	−0.0347 (18)	−0.0315 (16)
C25	0.100 (3)	0.0545 (18)	0.0569 (19)	0.0055 (18)	−0.0405 (19)	−0.0190 (15)
C26	0.081 (2)	0.076 (2)	0.0396 (16)	−0.0212 (18)	−0.0102 (16)	−0.0173 (15)
C27	0.0476 (16)	0.0667 (19)	0.0508 (16)	−0.0041 (14)	−0.0096 (14)	−0.0275 (14)
N31	0.0364 (11)	0.0353 (10)	0.0357 (10)	−0.0006 (8)	−0.0072 (9)	−0.0169 (9)
N32	0.0573 (15)	0.0457 (13)	0.0505 (13)	0.0041 (11)	−0.0010 (11)	−0.0263 (11)
C31	0.0421 (15)	0.0506 (15)	0.0452 (14)	−0.0046 (12)	−0.0048 (12)	−0.0230 (12)
C32	0.0621 (19)	0.0520 (16)	0.0474 (15)	−0.0075 (14)	−0.0044 (14)	−0.0299 (13)
C33	0.0401 (14)	0.0364 (13)	0.0419 (14)	0.0056 (11)	−0.0030 (12)	−0.0084 (11)
C34	0.0439 (16)	0.0628 (19)	0.0628 (19)	0.0138 (14)	−0.0055 (15)	−0.0205 (15)
C35	0.0355 (16)	0.084 (2)	0.065 (2)	0.0011 (15)	−0.0125 (15)	−0.0107 (17)
C36	0.0482 (17)	0.078 (2)	0.0540 (17)	−0.0079 (15)	−0.0188 (15)	−0.0183 (15)
C37	0.0422 (15)	0.0528 (16)	0.0447 (14)	−0.0024 (12)	−0.0100 (12)	−0.0189 (12)
C38	0.0356 (13)	0.0332 (12)	0.0331 (12)	0.0000 (10)	−0.0055 (10)	−0.0089 (10)

### Geometric parameters (Å, °)

**Table:**

Cu1—O12	1.9582 (15)	C23—C24	1.375 (3)
Cu1—O11	1.9660 (15)	C23—H23	0.9300
Cu1—O21	1.9735 (16)	C24—C25	1.367 (4)
Cu1—O22	1.9746 (16)	C24—H24	0.9300
Cu1—N31	2.2465 (18)	C25—C26	1.366 (4)
Cu1—Cu1^i^	2.6683 (6)	C25—H25	0.9300
O11—C11	1.258 (3)	C26—C27	1.383 (4)
O12—C11^i^	1.263 (2)	C26—H26	0.9300
C11—O12^i^	1.263 (2)	C27—H27	0.9300
C11—C12	1.498 (3)	N31—C31	1.313 (3)
C12—C13	1.377 (3)	N31—C38	1.370 (3)
C12—C17	1.383 (3)	N32—C32	1.302 (3)
C13—C14	1.383 (3)	N32—C33	1.366 (3)
C13—H13	0.9300	C31—C32	1.413 (3)
C14—C15	1.370 (4)	C31—H31	0.9300
C14—H14	0.9300	C32—H32	0.9300
C15—C16	1.371 (3)	C33—C34	1.415 (4)
C15—H15	0.9300	C33—C38	1.416 (3)
C16—C17	1.376 (3)	C34—C35	1.351 (4)
C16—H16	0.9300	C34—H34	0.9300
C17—H17	0.9300	C35—C36	1.395 (4)
O21—C21	1.255 (3)	C35—H35	0.9300
O22—C21^i^	1.267 (3)	C36—C37	1.369 (3)
C21—O22^i^	1.267 (3)	C36—H36	0.9300
C21—C22	1.500 (3)	C37—C38	1.407 (3)
C22—C23	1.381 (3)	C37—H37	0.9300
C22—C27	1.386 (3)		
			
O12—Cu1—O11	167.30 (6)	C27—C22—C21	120.9 (2)
O12—Cu1—O21	89.25 (7)	C24—C23—C22	121.2 (3)
O11—Cu1—O21	88.44 (7)	C24—C23—H23	119.4
O12—Cu1—O22	89.63 (7)	C22—C23—H23	119.4
O11—Cu1—O22	89.93 (7)	C25—C24—C23	120.2 (3)
O21—Cu1—O22	167.48 (6)	C25—C24—H24	119.9
O12—Cu1—N31	101.20 (7)	C23—C24—H24	119.9
O11—Cu1—N31	91.45 (6)	C26—C25—C24	119.5 (3)
O21—Cu1—N31	95.59 (7)	C26—C25—H25	120.3
O22—Cu1—N31	96.86 (7)	C24—C25—H25	120.3
O12—Cu1—Cu1^i^	85.49 (5)	C25—C26—C27	120.9 (3)
O11—Cu1—Cu1^i^	81.87 (5)	C25—C26—H26	119.6
O21—Cu1—Cu1^i^	85.08 (5)	C27—C26—H26	119.6
O22—Cu1—Cu1^i^	82.40 (5)	C26—C27—C22	120.0 (3)
N31—Cu1—Cu1^i^	173.27 (5)	C26—C27—H27	120.0
C11—O11—Cu1	125.83 (15)	C22—C27—H27	120.0
C11^i^—O12—Cu1	121.79 (15)	C31—N31—C38	116.26 (19)
O11—C11—O12^i^	125.0 (2)	C31—N31—Cu1	115.16 (15)
O11—C11—C12	117.4 (2)	C38—N31—Cu1	128.20 (14)
O12^i^—C11—C12	117.6 (2)	C32—N32—C33	115.7 (2)
C13—C12—C17	119.0 (2)	N31—C31—C32	122.6 (2)
C13—C12—C11	119.8 (2)	N31—C31—H31	118.7
C17—C12—C11	121.1 (2)	C32—C31—H31	118.7
C12—C13—C14	120.4 (2)	N32—C32—C31	123.1 (2)
C12—C13—H13	119.8	N32—C32—H32	118.5
C14—C13—H13	119.8	C31—C32—H32	118.5
C15—C14—C13	120.0 (2)	N32—C33—C34	119.2 (2)
C15—C14—H14	120.0	N32—C33—C38	122.0 (2)
C13—C14—H14	120.0	C34—C33—C38	118.9 (2)
C14—C15—C16	120.0 (2)	C35—C34—C33	120.1 (3)
C14—C15—H15	120.0	C35—C34—H34	119.9
C16—C15—H15	120.0	C33—C34—H34	119.9
C15—C16—C17	120.3 (2)	C34—C35—C36	121.1 (3)
C15—C16—H16	119.9	C34—C35—H35	119.4
C17—C16—H16	119.9	C36—C35—H35	119.4
C16—C17—C12	120.3 (2)	C37—C36—C35	120.6 (3)
C16—C17—H17	119.8	C37—C36—H36	119.7
C12—C17—H17	119.8	C35—C36—H36	119.7
C21—O21—Cu1	122.18 (16)	C36—C37—C38	119.8 (2)
C21^i^—O22—Cu1	125.03 (15)	C36—C37—H37	120.1
O21—C21—O22^i^	125.1 (2)	C38—C37—H37	120.1
O21—C21—C22	117.6 (2)	N31—C38—C37	120.2 (2)
O22^i^—C21—C22	117.2 (2)	N31—C38—C33	120.3 (2)
C23—C22—C27	118.2 (2)	C37—C38—C33	119.4 (2)
C23—C22—C21	120.8 (2)		

Symmetry codes: (i) −*x*+2, −*y*+1, −*z*+1.
